# Altered pupil light and darkness reflex and eye-blink responses in late-life depression

**DOI:** 10.1186/s12877-024-05034-w

**Published:** 2024-06-24

**Authors:** Yao-Tung Lee, Yi-Hsuan Chang, Hsu-Jung Tsai, Shu-Ping Chao, David Yen-Ting Chen, Jui-Tai Chen, Yih‐Giun Cherng, Chin-An Wang

**Affiliations:** 1 Department of Psychiatry, Shuang Ho Hospital, Taipei Medical University, New Taipei City, Taiwan; 2 Department of Psychiatry, Far Eastern Memorial Hospital, New Taipei City, Taiwan; 3 Department of Psychiatry, School of Medicine, College of Medicine, Taipei Medical University, Taipei, Taiwan; 4 Eye-Tracking Laboratory, Shuang Ho Hospital, Taipei Medical University, New Taipei City, Taiwan; 5 Institute of Cognitive Neuroscience, College of Health Science and Technology, National Central University, Taoyuan City, Taiwan; 6 Taipei Neuroscience Institute, Taipei Medical University, New Taipei City, Taiwan; 7 Dementia Center, Department of Neurology, Shuang Ho Hospital, Taipei Medical University, New Taipei City, Taiwan; 8 Department of Medical Image, Shuang Ho Hospital, Taipei Medical University, New Taipei City, Taiwan; 9 Department of Radiology, School of Medicine, College of Medicine, Taipei Medical University, Taipei City, Taiwan; 10 Department of Anesthesiology, School of Medicine, College of Medicine, Taipei Medical University, Taipei, Taiwan; 11 Department of Anesthesiology, Shuang Ho Hospital, Taipei Medical University, New Taipei City, Taiwan

**Keywords:** Pupillometry, Pupil light and darkness reflex, Autonomic function, Eye blink rate

## Abstract

**Background:**

Late-life depression (LLD) is a prevalent neuropsychiatric disorder in the older population. While LLD exhibits high mortality rates, depressive symptoms in older adults are often masked by physical health conditions. In younger adults, depression is associated with deficits in pupil light reflex and eye blink rate, suggesting the potential use of these responses as biomarkers for LLD.

**Methods:**

We conducted a study using video-based eye-tracking to investigate pupil and blink responses in LLD patients (*n* = 25), older (OLD) healthy controls (*n* = 29), and younger (YOUNG) healthy controls (*n* = 25). The aim was to determine whether there were alterations in pupil and blink responses in LLD compared to both OLD and YOUNG groups.

**Results:**

LLD patients displayed significantly higher blink rates and dampened pupil constriction responses compared to OLD and YOUNG controls. While tonic pupil size in YOUNG differed from that of OLD, LLD patients did not exhibit a significant difference compared to OLD and YOUNG controls. GDS-15 scores in older adults correlated with light and darkness reflex response variability and blink rates. PHQ-15 scores showed a correlation with blink rates, while MoCA scores correlated with tonic pupil sizes.

**Conclusions:**

The findings demonstrate that LLD patients display altered pupil and blink behavior compared to OLD and YOUNG controls. These altered responses correlated differently with the severity of depressive, somatic, and cognitive symptoms, indicating their potential as objective biomarkers for LLD.

**Supplementary Information:**

The online version contains supplementary material available at 10.1186/s12877-024-05034-w.

## Introduction

 Late-life depression (LLD) is a prevalent neuropsychiatric disorder in older adults, with an estimated prevalence ranging from 15 to 40% [1–4]. Despite LLD exhibiting high mortality rates from suicide and medical illness [5–7], it is often unrecognized and untreated in older adults because the symptoms of depression are frequently masked by pronounced physical health conditions. Therefore, it is essential to develop an easy-to-measure, objective method to facilitate the detection of LLD in the older population.

Pupil size is controlled by the balanced activity between the parasympathetic and sympathetic nervous systems [8–10], with pupil constriction in response to global luminance increase and pupil dilation in response to global luminance decrease [11, 12], known as the pupil light and darkness reflex (referred to as PLR and PDR), respectively. The PLR is primarily driven by parasympathetic activation, while PDR is mostly mediated by sympathetic activation [9, 13–15]. Examining the PLR and PDR can thus provide an assessment of autonomic functions. Correspondingly, the PLR has been widely used in clinical investigations [9, 16].

Autonomic nervous system dysfunction has been associated with depressive disorders in adults [17–19], as imbalanced autonomic functioning is commonly observed in psychiatric disorders [20–22]. Research has further highlighted autonomic dysfunction as a significant risk factor for depression [23]. However, the use of the PLR in the study of depression is still limited [24]. Research has generally shown that PLR responses have been attenuated in patients with depression compared to age-matched controls [25–27], though other effects have also been noted [25, 28]. This alteration is particularly sensitive to pupil responses to blue light [29–31]. Moreover, PLR responses can not only predict depression with suicidal risk [32, 33] but also predict the outcome with repetitive transcranial magnetic stimulation treatment [34]. While the PLR is a promising tool to study depression patients, it has yet to be used for the investigation in patients with LLD. Moreover, although response variability, commonly indexed by the coefficient of variation (CoV), provides insightful information for response performance [35] that is useful for clinical investigation (e.g [36]), previous PLR studies have not systematically examined response variability in depression patients.

LLD is a complex syndrome, and its pathophysiology involves multiple factors, affecting several neural systems [2]. Research in depression has been mostly focused on the PLR. While PDR, mediated mainly by the sympathetic pathway, could also provide clinical insight into individuals with depression, it is yet to be systematically investigated. Furthermore, tonic (baseline) pupil size, sought to reflect tonic neural activity of the locus coeruleus [37], and the locus coeruleus-norepinephrine (LC-NE) system is greatly influenced by age-related decline [38, 39]. Yet, research examining tonic pupil size in depression remains limited. Moreover, dysfunction of the dopaminergic system is noted in individuals with depression [40, 41]. Using eye blink rate to quantify central dopamine activity [42–45], research has shown higher blink rates in depression patients compared to controls [46–48]. However, all these measures have yet to be examined in LLD patients.

To investigate the function of the autonomic and dopaminergic systems in LLD patients, we employed video-based eye-tracking, measuring both pupil size and eye blinks. We systematically varied background luminance to induce both PLR and PDR responses in LLD patients, as well as in healthy younger and older adults. We hypothesized that light and darkness reflex, tonic pupil size, and eye blink rates should be altered in patients with LLD compared to healthy older and younger adults. More specifically, based on previous results in depression individuals, LLD patients should also exhibit attenuated PLR responses, and possibly reduced PDR responses, along with and higher eye blink rates compared to healthy controls. Regarding PDR and response variability, these aspects are subject to exploratory analysis without specific hypotheses.

## Methods and materials

### Experimental setup

All experimental procedures were reviewed and approved by the Institutional Review Board of the Taipei Medical University, Taiwan, and were in accordance with the Declaration of Helsinki [49]. Participants were naïve regarding the purpose of the experiment and provided informed consent with compensation for their participation. Twenty-five LLD patients, recruited from Shuang Ho Hospital by psychiatrist and co-author YL, participated in the study (mean age = 72 years, range: 61–81). Patients underwent assessments for somatic symptoms (Patient Health Questionnaire, PHQ-15), cognitive status (Montreal Cognitive Assessment, MoCA), and disease severity based on the Geriatric Depression Scale (GDS-15) [50–52]. We used the Chinese version of these neuropsychological tests, which have been previously validated [53–55]. Inclusion criteria focused on adults aged 65 years or older with a current DSM-5 diagnosis of nonpsychotic unipolar major depressive episode and the first lifetime depressive episode at age 65 or older. Participants were also required to be cognitively intact, without a clinical diagnosis of mild cognitive impairment or dementia. To exclude comorbid cognitive disorders, inclusion criteria included scores of the Mini-Mental State Examination (MMSE) [56] of 24 or above (for years of education > 6), 21 or above (for between 1 and 6 years of education), and 17 or above (for no education). Other common exclusion criteria included: (1) Current or past diagnoses of other psychiatric disorders, except for depression. (2) History of cognitive disorders, major neurological illnesses, and brain injuries. (3) Physically unstable patients. A comprehensive collection of correlated symptoms and signs, rather than structured interviews, was undertaken to confirm LLD cases. Final DSM-5 diagnoses were determined through diagnostic interviews conducted by co-author Y.L., a geriatric psychiatrist, and co-author S.C., a neurologist. Twenty-nine age-matched healthy older adults, with no history of major psychiatric disorders or neurological illnesses (mean age 73 years; range: 65–85), were also recruited (referred to hereafter as OLD). These participants were spouses or friends of the LLD participants or community members who responded to advertisements. Participants with comorbid neurological or ophthalmic conditions, such as macular degeneration or cataracts, were excluded. OLD did not significantly differ from LLD in terms of age, years of education, or MoCA scores. Additionally, twenty-five healthy younger adults (mean age 26 years; range: 20–35), referred to hereafter as YOUNG, were also recruited though advertisements. Three neuropsychological tests, except for MoCA, were not completed by one LLD participant and five OLD participants due to technical issues. These tests were not administered to YOUNG participants. Clinical data and participant demographics are presented in Table 1. LLD patients did not discontinue their medications for the study, adhering to approved institutional review board ethical guidelines. Table 2 displays medication characteristics, and antidepressants were categorized into six types, including serotonin/noradrenaline reuptake inhibitors (SNRI), selective serotonin reuptake inhibitors (SSRIs), norepinephrine-dopamine reuptake inhibitors (NDRI), tricyclic antidepressants (TCA), monoamine oxidase inhibitors (MAOI), and others. Sample sizes were determined based on our previous pupillometry studies in both healthy individuals and clinical populations [11, 57, 58].

**Table 1:** Demographics and clinical score of participants

Group	Number of Participants	Age at time of Measurement	Sex (male)	Education (years)	GDS15	PHQ15	MMSE	MoCA
LLD	25	72.2 ± 4.5	7	8.9 ± 3.5	10 ± 2.9	3.6 ± 3.9	26.4 ± 2.4	19.9 ± 4.4
OLD	29	72.8 ± 5.5	11	9.3 ± 3.8	1.5 ± 1.4	2.1 ± 2.9	27.3 ± 2.3	21.6 ± 3.7
YOUNG	25	24.6 ± 2.7	12	-	-	-	-	-

Mean ± SD. *LLD* late-life depression patients, *OLD* healthy age-matched older adults, *YOUNG* healthy younger adults, *GDS* Geriatric Depression Scale, *PHQ* Patient Health Questionnaire, *MMSE* Mental State Examination, *MoCA* Montreal Cognitive Assessment

**Table 2:** Medication of participants

Group	LLD	OLD
	*N* = 25	*N* = 29
SSRI	15	1
SNRI	1	0
NDRI	2	0
TCA	2	2
MAOI	0	0
Other antidepressants	3	0
α-blocker	1	5
β-blocker	8	5
Benzodiazepine	20	16
Anti-cholinergic	0	0
Anti-histamine	0	0

### Recording and apparatus

Participants were seated in a dark room, with an illuminance level of approximately 2.5 lx for 5 min to become familiar with the experiment setup and to listen to the instructions delivered by the experimenter. Eye position, pupil size and blink rate were measured with a video-based eye tracker (Eyelink-1000 plus binocular-arm, SR Research, Osgoode, ON, Canada) at a rate of 500 Hz with binocular recording, and stimulus presentation and data acquisition were controlled by the Eyelink Experiment Builder. Stimuli were presented on an LCD monitor at a screen resolution of 1920 × 1080 pixels with a 60 Hz refresh rate, subtending a viewing angle of 43° x 24°, with the distance from the eyes to the monitor set at 80 cm.

### Interleaved light and darkness reflex task (Fig. 1)

We used the light and darkness reflex task [58] to compare pupil light and darkness reflex responses between the three groups. Each trial began with the appearance of a central fixation point (FP) (0.5° diameter, 25 cd/m2; referred to hereafter as cd/m2) on a gray background (10 cd/m2). After 900–1100 ms of central fixation, background luminance either increased to 15–20 cd/m^2^, decreased to 0.1–5 cd/m^2^ (both with 50 and 100% contrast relative to the gray background), or stayed the same (10 cd/m^2^). Participants were required to maintain steady fixation for an additional 2–2.5 s. The next trial commenced after an inter-trial interval of 3–4 s. Background luminance conditions were randomly interleaved, and each condition had 35 trials in the LLD and OLD groups, and had 20 trials in the YOUNG group trials, lasting approximately 25 and 18 min, respectively.

**Fig. 1:**
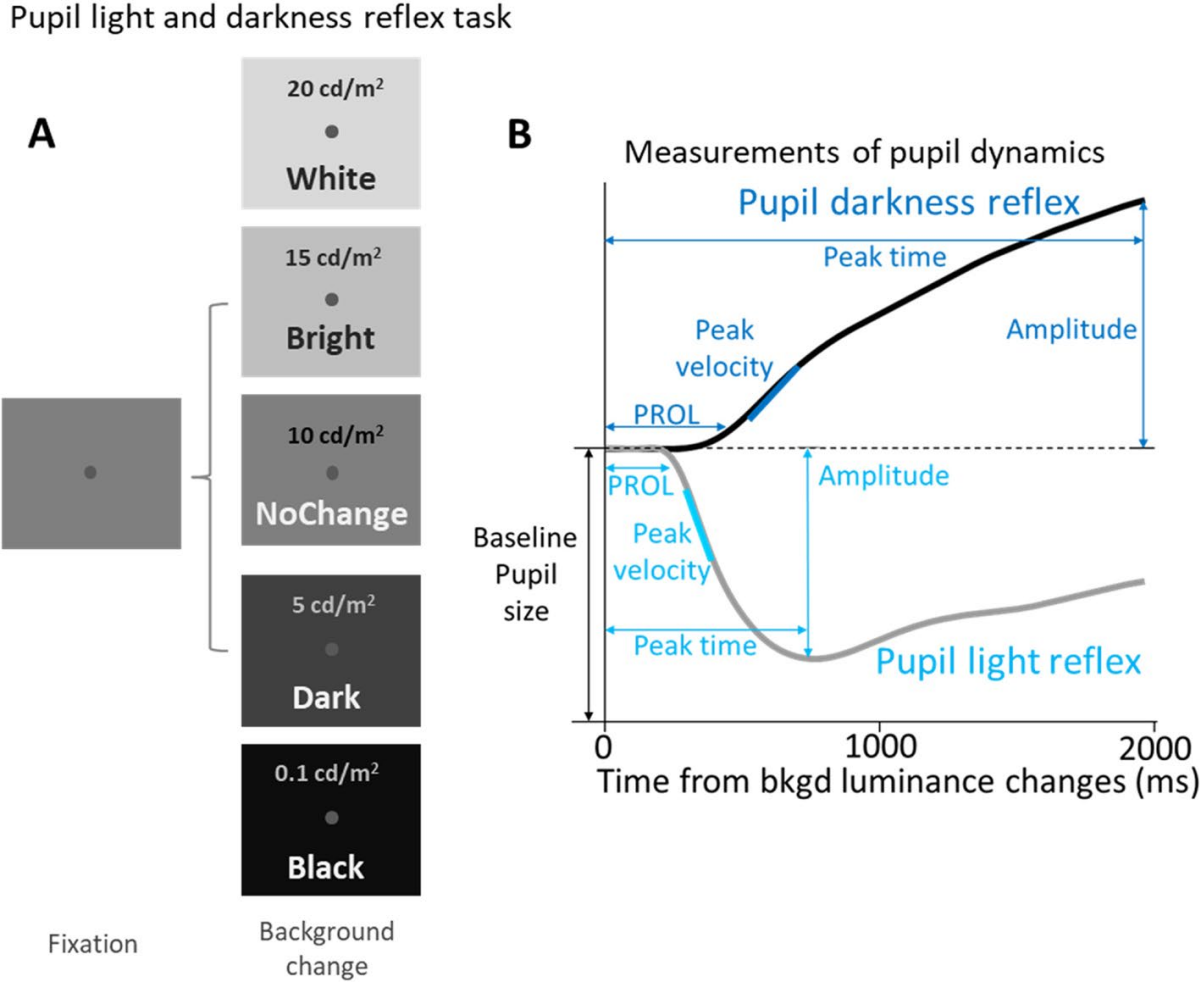
**A** Experimental paradigm. Each trial started with a central fixation point on a gray background. After a delay, the background luminance either increased (20–15 cd/m^2^), decreased (5–0.1 cd/m^2^), or stayed the same (10 cd/m^2^). Participants were required to maintain steady fixation for an additional 2000–2500 ms. **B** Measurements of pupil response dynamics. PROL: pupil response onset latency. Peak Velocity: peak pupil response velocity. Amplitude: peak pupil response size. Peak Time: time to peak response. Bkgd: background

### Data analysis

To maintain an accurate measure of pupil size, participants were required to maintain central fixation during the task. The pupil responses are consensual [9, 11] and we arbitrarily selected the data from the left eye for analysis because it usually showed higher accuracy in our previous data collection experience. Following the proposed procedure [59], we used the available MATLAB codes for pupil data preprocessing to remove invalid data time points, and pre- and post-invalid pupil values were used to perform a linear interpolation to replace invalid pupil values. After that, the data were smoothed using a zero-phase low-pass filter with a cut-off frequency of 5 Hz, because our previous research, alongside others, demonstrates that pupil oscillations primarily occur at frequencies below 5 Hz [60]. To investigate task-evoked responses, a baseline-correction procedure was used [61]. The baseline pupil size for each trial was determined by averaging pupil size from 200 ms before to the onset of the background luminance change. We then subtracted this baseline value from original pupil values. Because pupil size was constantly changing even when there was no stimulus presented and, to simplify data presentation and quantification, we normalized pupil diameter values by contrasting the background change versus no-background-change conditions directly [62]. Specifically, pupil values from each background change trial were contrasted to the average pupil value from all control trials. Because the tonic pupil size (pre-baseline-correction) is hypothesized to reflex tonic LC activity [37], we quantified tonic pupil size in the mean absolute pupil size in the baseline epoch (-200 to 0 ms of background luminance change onset). Because pupil light and darkness reflexes are primarily driven by the parasympathetic and sympathetic system, respectively [9, 13–15], two time windows were arbitrarily selected to separately capture pupil light and darkness peak responses: (1) an epoch spanning from 600 to 900 ms after the background change onset was selected for the pupil light reflex because the time to peak constriction was ~ 754 ms; and (2) an epoch spanning from 1900 to 2000 ms was selected for the pupil darkness reflex because the time to peak dilation was ~ 2000 ms. For each subject, coefficient of variation (CoV) (standard deviation/mean × 100) was also computed to measure response variability. Because CoV is generally calculated using non-negative values and pupil constriction are considered as negative values, we added the absolute value of the minimum value (the most negative value) plus a small constant value (to avoid division by zero) to all data points. This procedure ensured that all values were positive. We additionally measured eye blinks using previously developed algorithms [59] because eye blinks are not only linked to cognitive load (e.g [63]). . but also associated with dopaminergic activity [64]. Eye blinks are typically categorized into three types: spontaneous, reflex, and voluntary, with spontaneous eye blinks specifically linked to dopamine activity [64, 65]. In our study, participants were simply required to maintain central fixation, and the nature of the stimuli did not induce eye blinks. Therefore, the eye blinks measured here should be considered as spontaneous eye blinks. Moreover, while eye blink rates are influenced by various viewing factors, such as reading from a computer screen compared to reading from a hard copy [66, 67], this influence would be consistent across all three groups in our study. Eye blink rate around the onset of background luminance change (-1 to 2 s) was calculated to indirectly measure dopamine level [42–45]. Note that outlier values in baseline pupil size beyond 1.5 times the interquartile range (the difference between upper and lower quartiles) below the lower quartile or above the upper quartile were excluded from analysis. The above criteria resulted in the removal of 6.95% of trials.

Pupil metrics were analyzed [9, 16, 68–71], and similar to our previous research [72, 73], four pupil indices were reported (Fig. 1B). We first calculated pupil response onset latencies (PROL) that were defined as the time point at which pupil acceleration reached its maximal and pupil velocity was negative (i.e. constricting) in the pupil light reflex conditions (or positive in the pupil darkness reflex conditions) according to the established criteria [74]. Moreover, we calculated the maximum response amplitude, and the maximum response velocity of the pupil response. Additionally, we calculated the time of maximum responses for the time that pupil size reached its maximal constriction or dilation (referred to as Peak Time).

A mixed ANOVA (3 × 4 ANOVA: between-subjects factor: LDD/OLD/YOUNG × within-subjects factor: background luminance level) was performed for statistical analysis with a Tukey’s HSD post hoc comparison unless stated otherwise, and homogeneity correction was applied where necessary. The simple main effect was further used to specifically test our hypothesis that the modulation of pupil responses was different among three groups. A one-way ANOVA was used for tonic pupil size and blink rate analyses. Correlational analyses were further performed to examine the relationship between scores of neuropsychological tests and pupil measures in LLD and OLD participants, so we collapsed these two group participants. Tonic pupil size, pupil light and darkness reflex in lower contrast conditions (bright and dark), and eye blink rates were selected to examine their correlations with the scores of neuropsychological tests. We used the bright and dark conditions to avoid the ceiling effect that may be resulted from using a high contrast of background luminance change. All statistical comparisons were performed using JASP Team [75] and MATLAB (The MathWorks Inc., Natick, MA, USA).

## Results

### Tonic pupil size

**Fig. 2:**
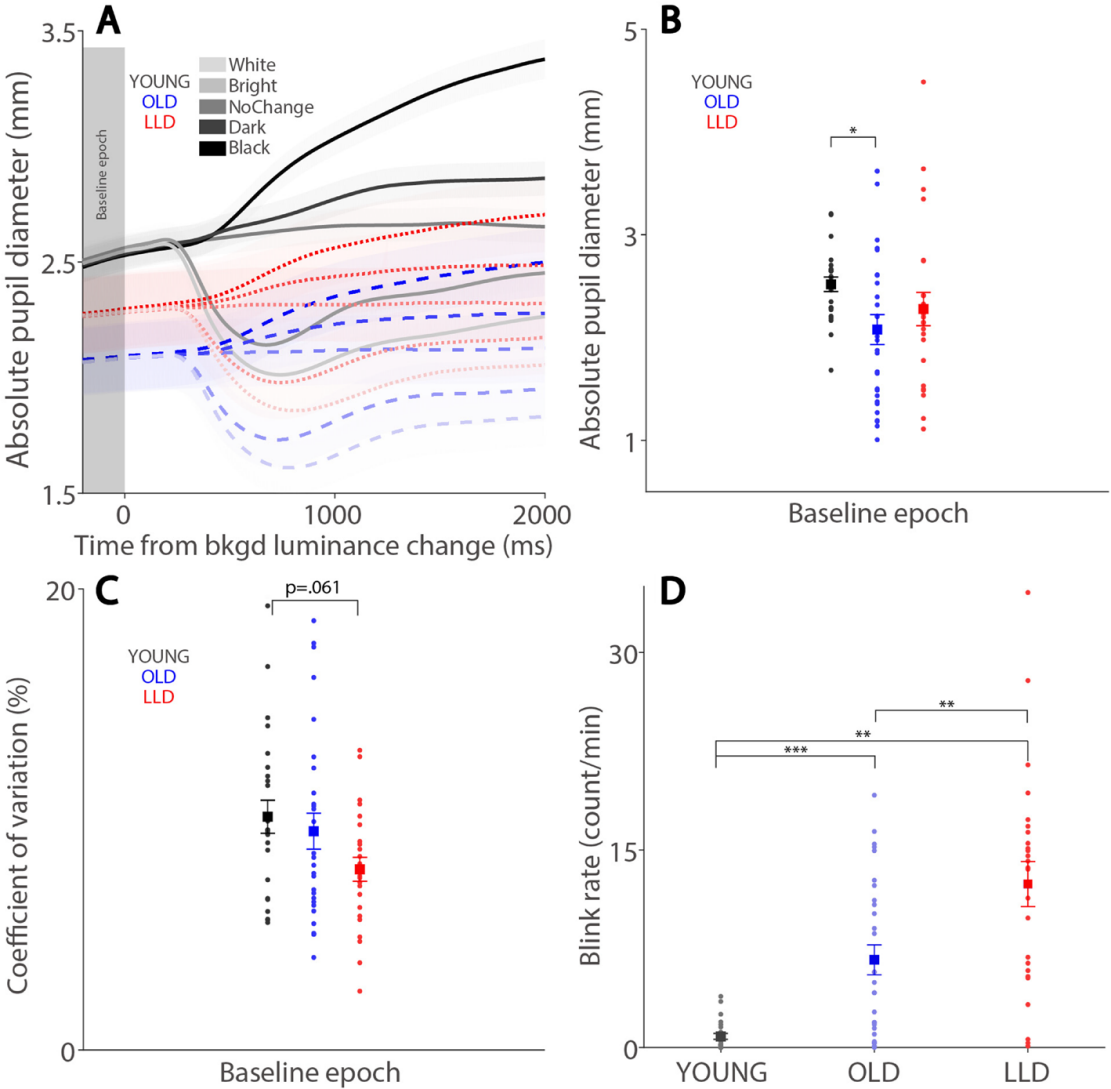
Tonic pupil size and blink rate effects for each experimental group. Dynamics of absolute pupil diameter following background luminance change in different conditions (**A**). Mean pupil sizes (tonic pupil size) at the baseline epoch (-200 to 0 ms) (**B**), mean coefficient of variation of tonic pupil size (**C**), and eye blink rate (**D**) shown for different experimental groups. In **A**, the shaded colored regions surrounding the pupil response curves represent the ± standard error range (across participants) for different conditions. The gray area represents the epoch selected for tonic pupil size analyses. In **B**-**D**, the large-squares and error-bars represent the mean values ± standard error across participants. The small circles represent the mean value for each participant. White: background luminance 20 cd/m^2^. Bright: background luminance 10 cd/m^2^. NoChange: background luminance 10 cd/m^2^ (stayed the same). Dark: background luminance 5 cd/m^2^. Black: background luminance 0.1 cd/m^2^. healthy younger adults. YOUNG: healthy younger adults. OLD: healthy age-matched older adults. LLD: late-life depression patients. Bkgd: background. *P ≤ 0.05, **P ≤ 0.01, ***P ≤ 0.001

We first examined the effect of tonic pupil size in the three groups, Fig. 2A shows dynamics of absolute pupil size relative to the change of background luminance. All background luminance conditions were then collapsed to investigate absolute pupil sizes before the luminance change. Mean pupil sizes at the baseline epoch (-200 ms to luminance change onset, see “Methods and materials” section) were significantly different between groups (F(2,76) = 4.242, *p* = 0.021, η_p_^2^ = 0.070) (Fig. 2B), showing smaller tonic pupil sizes in older individuals. Post hoc comparison of groups determined that significantly smaller pupil sizes in OLD compared to YOUNG participants (*p* = 0.049). To examine variability of tonic pupil size, coefficient of variation (CoV) shows significant differences among the three groups (F(2,76) = 4.242, *p* = 0.021, η_p_^2^ = 0.070) (Fig. 2C). Post hoc comparison revealed a lower CoV in LLD compared to YOUNG participants, though these differences were only approached significance (*p* = 0.061).

### Blink rate

There was a significant difference in blink rate between the three groups during the task-related interval (-1 to 2 sec relative to background luminance change onset) (F(2,76) = 35.054, *p* < 0.001, η_p_^2^ = 0.381) (Fig. 2D). Post hoc pairwise comparison of groups determined that LLD (*p* = 0.002) and OLD (*p* < 0.001) participants made significantly more blinks than YOUNG participants. Moreover, there were also significant higher blink rates in LLD compared with OLD participants (*p* = 0.002).

### Pupil light and darkness reflex

**Fig. 3:**
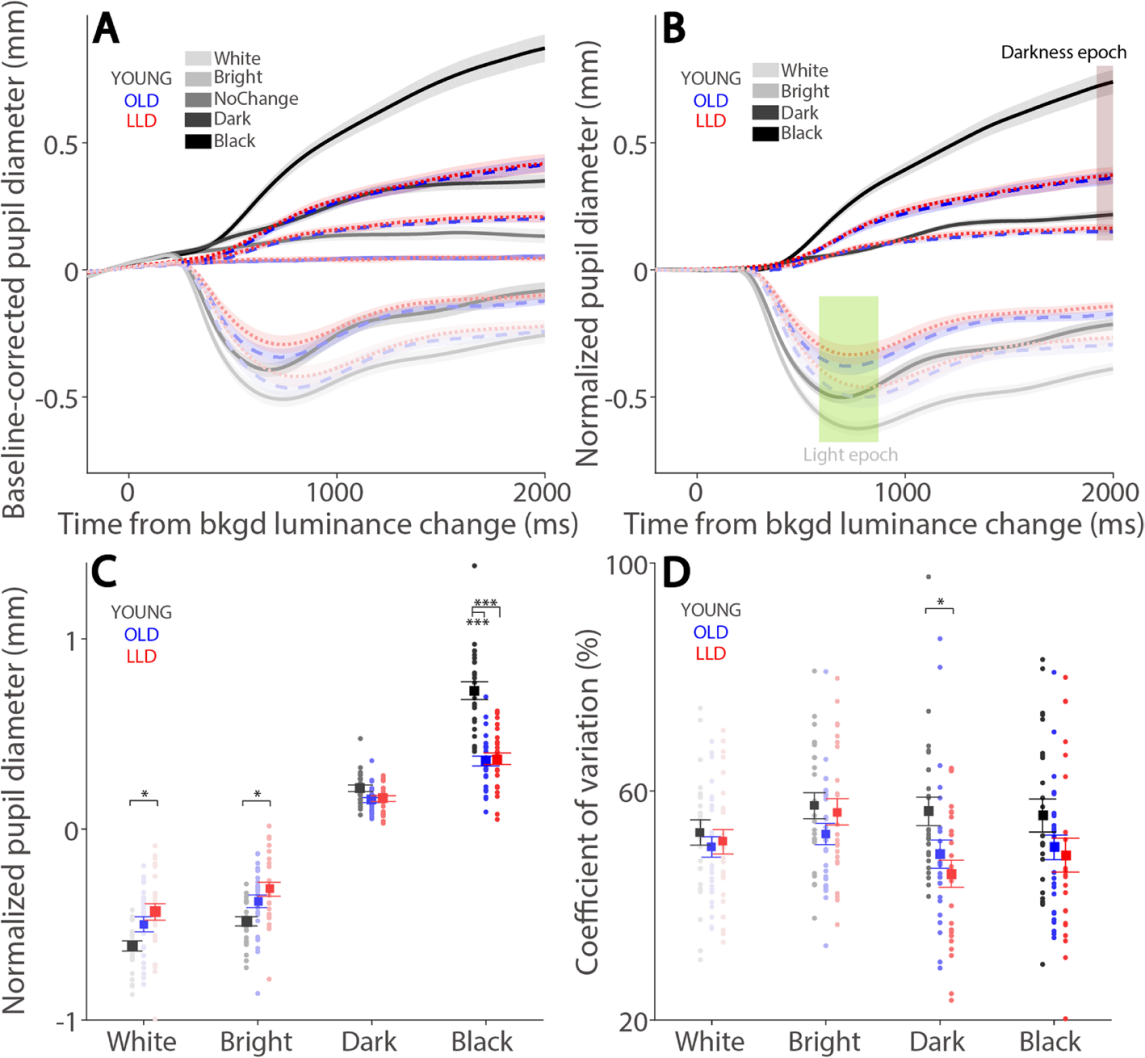
Pupil light and darkness reflex responses for each experimental group. Dynamics of baseline-corrected pupil diameter following background luminance change in different conditions (**A**). Normalized pupil light and darkness responses (light/darkness responses minus no change responses) (**B**). Mean pupil sizes at the light (600-900ms) or darkness (1900-2000ms) epoch (**C**), and mean coefficient of variation of PLR and PDR pupil size (**D**) shown for different conditions and experimental groups. In **A**-**B**, the shaded colored regions surrounding the pupil response curves represent the ± standard error range (across participants) for different conditions. In **B**, the light and darkness epochs for pupil size are shaded in colors. In **C-D**, the large-squares and error-bars represent the mean values ± standard error across participants. The small circles represent the mean value for each participant. White: background luminance 20 cd/m^2^. Bright: background luminance 10 cd/m^2^. NoChange: background luminance 10 cd/m^2^. Dark: background luminance 5 cd/m^2^. Black: background luminance 0.1 cd/m^2^. healthy younger adults. YOUNG: healthy younger adults. OLD: healthy age-matched older adults. LLD: late-life depression patients. Bkgd: background. *P ≤ 0.05, **P ≤ 0.01, ***P ≤ 0.001

As displayed in Fig. 3A, changes in background luminance resulted in transient pupil responses (baseline-corrected), with pupil constriction and dilation in response to luminance increase and decrease, respectively, as documented in the literature [9, 11–13]. Notably, pupil size changed even without changes in background luminance (Fig. 3A). To normalize pupil size, we contrasted the luminance change condition to the no luminance change condition in each group separately, as illustrated in Fig. 3B (see “Methods and materials” section). To further quantify these results, the light epoch (600–900 ms) and darkness epoch (1900–2000 ms) were used (see “Methods and materials” section). Pupil responses were as a function of background luminance changes (F(3,228) = 834.234, *p* < 0.001, η_p_^2^ = 0.917) (Fig. 3C), and the size of pupil responses scaled with change contrast of background luminance (all *p* < 0.001 with a Holm post hoc pairwise comparison). Moreover, there was a significant interaction between groups and background luminance conditions (F(6,228) = 23.209, *p* < 0.001, η_p_^2^ = 0.379). The simple main effects revealed significant differences between groups in all luminance conditions (all *p* < 0.01). Specifically, larger pupil constriction and dilation were often observed in YOUNG compared to OLD participants, and these differences were more pronounced in the light reflex compared to the darkness reflex conditions. Furthermore, LLD patients showed reduced pupil light reflex responses compared to OLD participants, though this effect was not significant in post hoc comparison. As displayed in Fig. 3D, there was a significant difference between background luminance conditions in CoV (F(3,228) = 3.318, *p* = 0.024, η_p_^2^ = 0.042). Holm post hoc pairwise comparison of groups revealed that Dark (*p* = 0.049) and Black (*p* = 0.049) conditions had significantly lower CoV than the bright condition. Moreover, there was a significant difference between groups (F(2,76) = 5.026, *p* = 0.009, η_p_^2^ = 0.117). Post hoc comparison of conditions determined that OLD participants had significantly lower CoV than YOUNG participants (*p* = 0.026). Moreover, there was no interaction between groups and background luminance conditions (F(6,228) = 1.572, *p* = 0.163, η_p_^2^ = 0.040).

### Pupil metrics

**Fig. 4:**
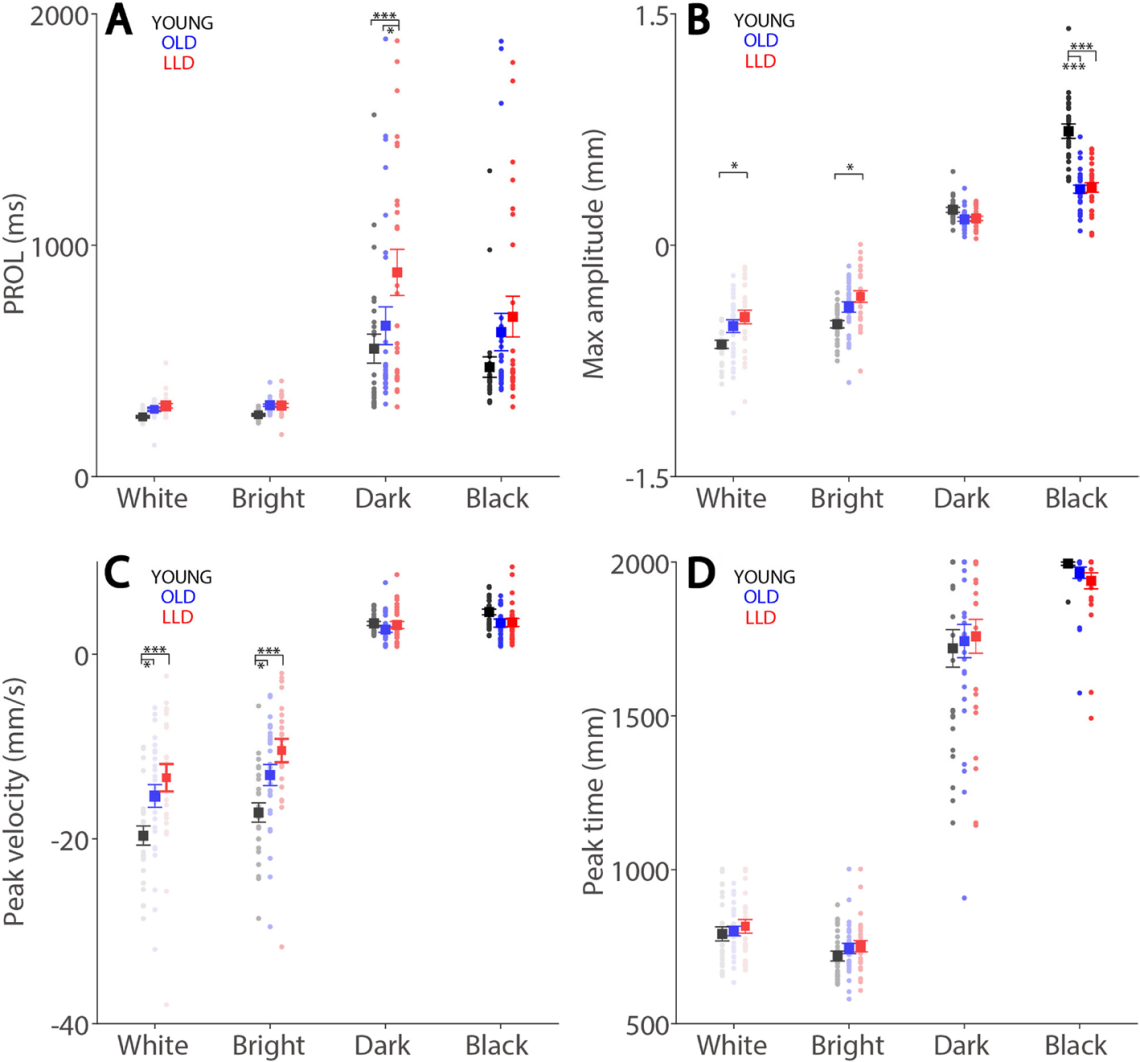
Pupil metrics for each experimental group. Mean PROL (**A**), max response amplitude (**B**), max response peak velocity (**C**), and time to peak response (**D**) shown for different conditions and experimental groups. In **A-D**, the large-squares and error-bars represent the mean values ± standard error across participants. The small circles represent the mean value for each participant. White: background luminance 20 cd/m^2^. Bright: background luminance 10 cd/m^2^. Dark: background luminance 5 cd/m^2^. Black: background luminance 0.1 cd/m^2^. healthy younger adults. YOUNG: healthy younger adults. OLD: healthy age-matched older adults. LLD: late-life depression patients. PROL: pupil response onset latency. Peak Velocity: peak pupil response velocity. Max amplitude: peak pupil response size. Peak Time: time to peak response. *P ≤ 0.05, **P ≤ 0.01, ***P ≤ 0.001

Consistent with the literature [9, 11–13], the PROL (pupil response onset latency) (Fig. 4A) was significantly faster in the light compared to the dark condition, with shorter PROL in the higher contrast conditions regardless of luminance change polarity (background luminance main effect: F(3,228) = 51.528, *p* < 0.001, η_p_^2^ = 0.404), with 286, 295, 700, and 598 ms for the white, bright, dark, and black condition, respectively. Moreover, there was a significant interaction between groups and background luminance conditions (F(6,228) = 2.443, *p* = 0.049, η_p_^2^ = 0.060). The simple main effects revealed significant differences between groups in all luminance conditions (all *p* < = 0.011) except for the black condition (*p* = 0.097). Post hoc comparison of groups determined that YOUNG (*p* < 0.001) and OLD (*p* = 0.036) participants had significantly faster PROLs than LLD participants in the dark condition. In max amplitude (Fig. 4B), there was a significant difference between conditions (F(3,228) = 904.295, *p* < 0.001, η_p_^2^ = 0.922), with larger constriction or dilation amplitudes with higher contrast conditions. A significant interaction between groups and conditions was obtained (F(6,228) = 24.250, *p* < 0.001, η_p_^2^ = 0.390). Moreover, the simple main effects further revealed significant differences in the all conditions (all *p* < = 0.008). Post hoc comparison of groups determined that YOUNG participants had significantly larger constriction than OLD participants in the white (*p* = 0.015) and bright (*p* = 0.010) conditions. Additionally, YOUNG participants had significantly larger pupil dilation than OLD (*p* < 0.001) and LLD (*p* < 0.001) participants in the black condition.

In peak velocity (Fig. 4C), there was a significant difference between conditions (F(3,228) = 608. 301, *p* < 0.001, η_p_^2^ = 0.889), with larger constriction or dilation velocities with higher contrast conditions. A significant interaction between groups and conditions was obtained (F(6,228) = 7.663, *p* < 0.001, η_p_^2^ = 0.168). Furthermore, the simple main effects revealed significant differences in the white (*p* = 0.004) and bright (*p* < 0.001) conditions. Post hoc pairwise comparison of groups determined that LLD and OLD participants made significantly smaller constriction than YOUNG participants in the white (OLD: *p* = 0.012; LLD: *p* < 0.001) and bright conditions (OLD: *p* = 0.021; LLD: *p* < 0.001). In peak time (Fig. 4D), there was a significant difference between conditions (F(3,228) = 1303.528, *p* < 0.001, η_p_^2^ = 0.945). Holm post hoc comparison of conditions revealed significant differences between all conditions (all *p* < 0.001), showing larger peak times in the higher contrast conditions regardless of luminance change polarity. All other effects were not significant.

### Correlations between neuropsychological test scores and pupil and eye blink rate measures

**Fig. 5:**
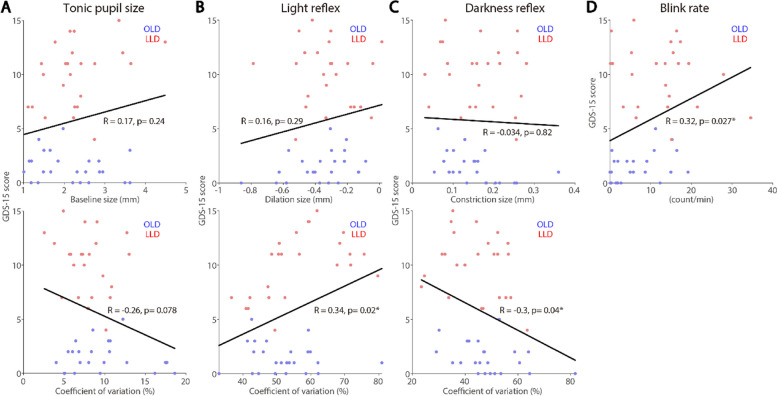
Inter-individual correlations between pupil and blink responses and GDS-15 scores. Correlation between GDS-15 scores and tonic pupil size (**A**), light reflex responses (**B**), light reflex responses (**C**), eye blink rate (**D**). OLD: healthy age-matched older adults. LLD: late-life depression patients. GDS: Geriatric Depression Scale

**Fig. 6:**
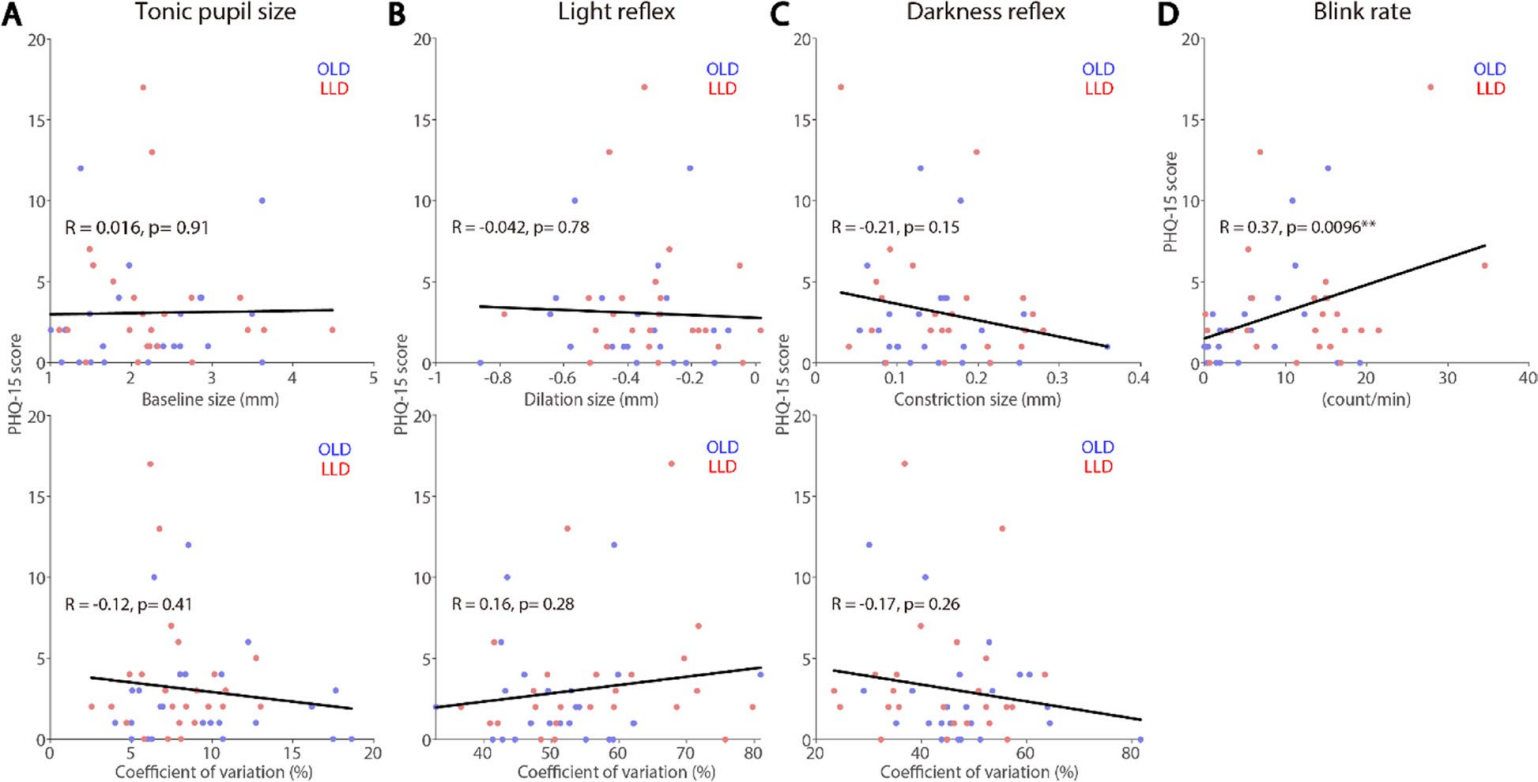
Inter-individual correlations between pupil and blink responses and PHQ-15 scores. Correlation between PHQ-15 scores and tonic pupil size (**A**), light reflex responses (**B**), light reflex responses (**C**), eye blink rate (**D**). OLD: healthy age-matched older adults. LLD: late-life depression patients. PHQ: Patient Health Questionnaire

**Fig. 7:**
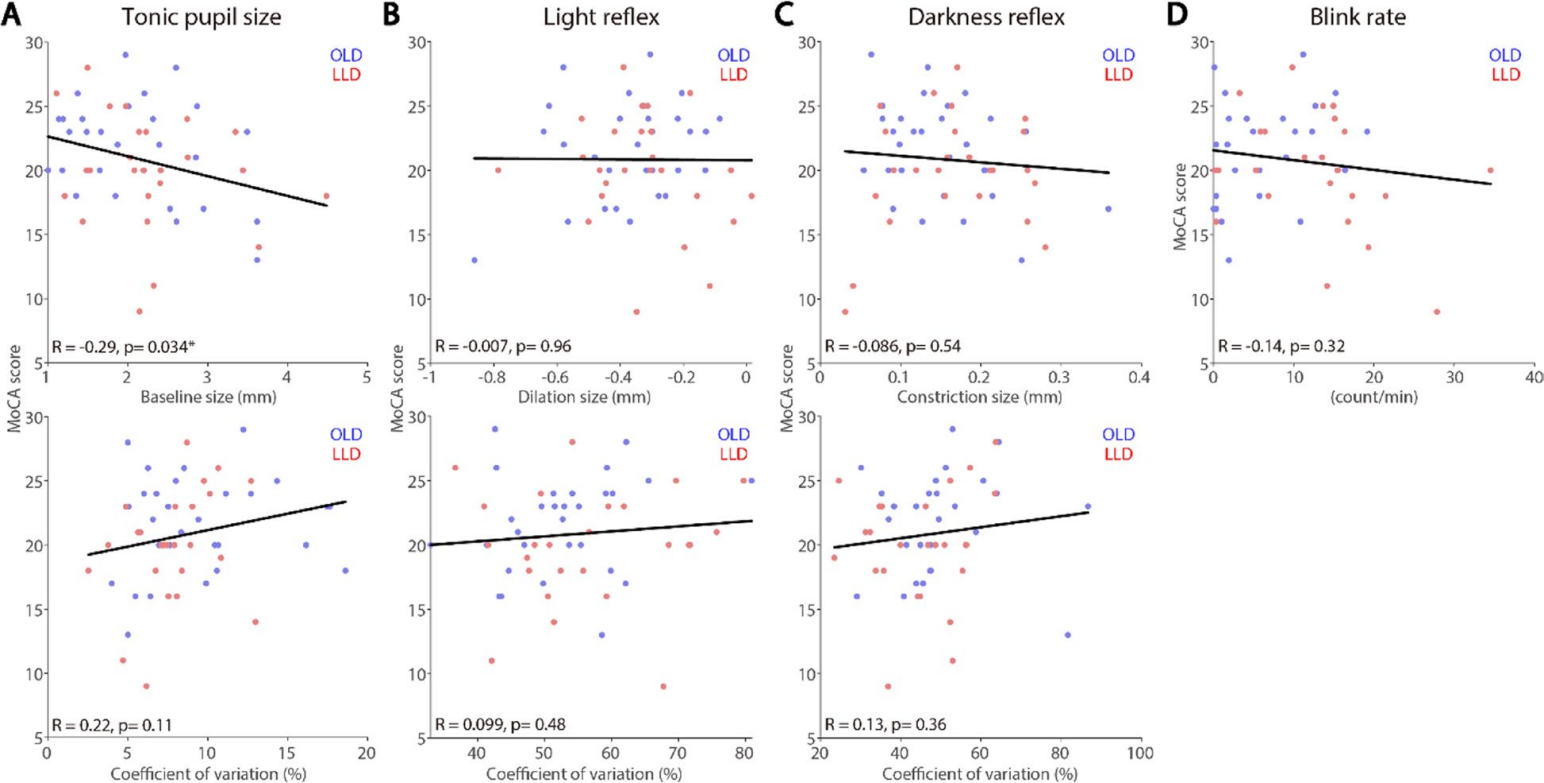
Inter-individual correlations between pupil and blink responses and MoCA scores. Correlation between MoCA scores and tonic pupil size (**A**), light reflex responses (**B**), light reflex responses (**C**), eye blink rate (**D**). OLD: healthy age-matched older adults. LLD: late-life depression patients. MoCA: Montreal Cognitive Assessment

The relationship between neuropsychological tests scores and pupil and blink measures was examined. GDS-15 and PHQ-15 were used to respectively assess their disease severity and somatic symptoms, and MoCA was used to assess their cognitive function. LLD and OLD participants were collapsed, such that we can examine whether pupil and eye measures could correlate with these scores in ageing population, focusing on tonic pupil size, light and darkness reflex sizes, and blink rate (see “Methods and materials” section). As illustrated in Fig. 5, scores on the GDS-15 significantly correlated with light reflex CoV (Fig. 5B, *R* = 0.34, *p* = 0.02), darkness reflex CoV (Fig. 5C, *R* = 0.3, *p* = 0.04), and blink rate (Fig. 5D, *R* = 0.32, *p* = 0.027), showing severe depression symptoms correlating to higher light reflex CoV, lower darkness reflex CoV and higher blink rates. In contrast, GDS-15 scores did not correlate with tonic pupil and darkness reflex responses. Figure 6D illustrates a positive correlation between PHQ-15 score and blink rates (*R* = 0.37, *p* < 0.001), showing a more severe levels of somatization correlating with higher blink rates. Scores on the PHQ-15 did not correlate with other pupil measures (Fig. 6A-C). As displayed in Fig. 7, tonic pupil size significantly correlated with MoCA scores (Fig. 7A, *R* = 0.29, *p* = 0.034), with higher pupil sizes correlating with lower scores. MoCA scores did not correlate with other measures (Fig. 7B-D).

## Discussion

To investigate autonomic and dopaminergic functions in Late-Life Depression (LLD) patients, we analyzed the dynamics of pupil light and darkness reflex, as well as tonic pupil size and eye blink rates in a task with varying background luminance to evoke PLR and PDR in LLD, younger, and older healthy participants. Tonic pupil sizes differed between groups, with significantly larger pupil sizes in YOUNG compared to OLD. Moreover, eye blink rates varied significantly between the three groups, with lower rates in both YOUNG and OLD compared to LLD. Furthermore, light reflex responses differed between groups, with greater reflex responses in YOUNG compared to LLD. Although differences between LLD and OLD were noted, they were not statistically significant. In darkness reflex, while reflex sizes were larger in YOUNG compared to OLD and LLD, there were generally no significant differences between LLD and OLD. Additionally, depression symptom severity correlated with light reflex and darkness response variabilities and blink rates. In contrast, somatic symptom severity only correlated with blink rates. MoCA scores correlated with tonic pupil sizes. Overall, our findings demonstrate altered pupil and blink rate responses in LLD compared to OLD and YOUNG, highlighting the value of analyzing all these measures to gain insights into the impact of depressive symptoms on older adults.

### Pupil light and darkness reflex and autonomic function

The PLR is an effective tool for studying autonomic functions in the diseased population [9, 16]. While autonomic dysfunction in individuals with depression has been noted [17–19], no prior studies have identified altered PLR or PDR in LLD. Here, we examined both PLR and PDR in LLD as well as in YOUNG and OLD. Consistent with the literature [13], PLR and PDR induced by background luminance changes responded more significantly and rapidly in YOUNG compared to OLD. Additionally, the change intensity of background luminance systematically affected PLR and PDR responses, with more robust responses obtained in the high-contrast change condition (i.e., 100% contrast). Importantly, trends of differences between LLD and OLD were observed, with smaller responses in LLD compared to OLD, although they often did not survive multiple comparisons. These results were generally consistent with findings in younger depressed adults [25–27, 29, 30, 76], showing attenuated PLR-related responses in depression individuals. Furthermore, consistent with this idea, patients with larger improvements after repetitive-TMS treatment exhibit larger pupil constriction amplitudes [34]. Moreover, larger differences between LLD and OLD were often noted in the low-contrast background luminance condition (i.e., 50% contrast). In line with previous research [30], this information informed subsequent research to consider the possibility of a ceiling effect implemented by a larger luminance change intensity. Furthermore, light and darkness reflex response variability indexed by the Coefficient of Variation (CoV) correlated with the scores of GDS-15, suggesting the value of analyzing response variability to reveal depression symptom severity.

While depression individuals often exhibit attenuated PLR-related responses [25–27, 29, 30, 76, 77], other effects have also been noted. Some studies report no differences in PLR-related responses between individuals with depression and healthy controls [28]. Shorter PLR response latencies are noted in individuals with depression compared to healthy controls [25]. Moreover, a recent study found larger PLR responses in depression patients with suicide risk compared to non-suicidal depressed patients and healthy controls [32], and hyperarousal is associated with suicidal ideation in depression patients [33]. These results together suggest that individuals with depression may generally exhibit attenuated PLR-related responses, however, those with suicide risk may display more rigorous PLR responses, indicating a state of hyperarousal. Because a previous study was conducted with a small cohort [32], future studies are certainly needed to further investigate this line of research. Overall, pupil light and darkness reflexes were modulated by age, and more blunted responses were seen in patients with LLD.

### Tonic pupil size and the locus coeruleus-norepinephrine system

Tonic pupil size (baseline) has been associated with a tonic firing mode of LC activity [37]. The LC is a major nucleus that releases norepinephrine throughout most of the brain via its extensive network of axons axons [37, 78]. Research has shown that the LC-NE system changes as a function of age, and the relationship between LC-NE changes and cognitive decline has been consistently noted [38, 39, 79, 80]. Consistent with this idea, using tonic pupil size to indirectly index tonic LC activity, we found smaller baseline pupil sizes in OLD compared to YOUNG, consistent with previous research [13]. Interestingly, Montreal Cognitive Assessment (MoCA) scores, as an index of cognitive function, negatively correlated with tonic pupil size in older individuals, with smaller, not larger, pupil sizes correlating with higher MoCA scores. These results were not in line with our expectations, and this could be due to altered responses in LLD, as baseline pupil size seemed to be larger in LLD compared to OLD, although these differences were not statistically significant. Future investigations are necessary to test these observed correlations.

### Eye blink rate and the dopaminergic system

While deficits in the dopaminergic system have been noted in depression patients [40, 41], its influences on LLD are more complex and require more thorough investigation [81]. Here, we used spontaneous eye blink rate to indirectly measure central brain dopamine activity [42–45, 64]. Consistent with previous studies in depressed adults (Mackintosh et al., 1983; Ebert et al., 1996; Byrne et al., 2016), LLD exhibited significantly higher blink rates compared to YOUNG and OLD. These results provide evidence suggesting deficits in the dopaminergic system in LLD. Moreover, scores of Patient Health Questionnaire-15 (PHQ-15) and GDS-15 positively correlated with blink rates in older participants, suggesting a functional role of this deficit in correlating with depression and somatic symptom severity. Notably, spontaneous eye blink rates are modulated by diurnal variation [82]. While participants here were collected from 9 am to 4 pm, and spontaneous eye blink rates should be similar in these time periods [82], future investigations are certainly needed to take this factor into consideration.

### Medication effects on eye blink and pupil responses

LLD patients recruited were not discontinued from their medication, and both LLD patients and OLD controls were indeed taking different medications. It is thus important to consider these medications as potential variables in our analyses because, as shown previously, some medications such as SNRIs can affect pupil responses [83]. Moreover, previous research has argued attenuated PLR responses in depression individuals are mediated by medication [77]. To control for medication influences, we used linear mixed models, allowing us to include medication type as a fixed factor in addition to the effects of patient group, focusing on LLD and OLD groups. As illustrated in Additional file 1, significant (or trending) differences between LLD and OLD persist in tonic pupil size variability, blink rates, and pupil response onset latencies for the darkness reflex even after taking medication type into account. Furthermore, other drugs, as displayed in Table 2, that can potentially affect the autonomic nervous system were also taken into consideration. As shown in Additional file 2, blink rates were significantly influenced by beta blockers and benzodiazepine. However, more importantly, the group effects remain unchanged after accounting for these drugs. These results together suggest that the observed differences between LLD and OLD cannot be solely explained by the effects of medication.

### Limitations and future directions

The current paradigm allowed us to investigate the parasympathetic and sympathetic function among the LLD, OLD, and YOUNG groups by systematically varying the background luminance level. However, future work would benefit from simplifying the luminance change conditions to increase statistical power for further explorations in this line of research. Moreover, many exploratory analyses were conducted without a primary hypothesis, increasing the risk of Type I error, and the absence of a structured diagnostic interview is certainly a limitation. Furthermore, older participants may experience vision-related issues, which could potentially affect their task performance. While these participants have no history of eye-related diseases and their vision, indirectly measured by a sub-item Construction in MMSE, was normal, it is advisable to involve an ophthalmologist in future investigations to ensure that participants do not have ocular diseases such as cataracts and macular degeneration. Note that the current study did not conduct urine drug screening for nicotine and cannabis use, and this needs to be taken into account for future studies. Furthermore, research has suggested a 20-minute period of dark adaptation for consistent pupil constriction responses [84]. Future research should also consider this factor to obtain a more reliable PLR response. Moreover, while the sympathetic function can be measured by the PDR, this function is arguably measured more effectively using affectively salient stimuli [85]. Future work using emotional stimuli is needed to examine sympathetic function in LLD. Additionally, while some disruptions of PLR modulations were observed here, some previous studies have shown larger effects in pupil responses to blue light in depression patients [28–31], warranting further investigation using blue light. Finally, the current study is limited by the characterization of altered pupil and eye blink responses in a small study cohort consisting of only younger and older adults. Future work with larger study cohorts is certainly needed to investigate these altered pupil and eye blink responses in LLD.

## Conclusions

Pupil light and darkness reflex have been extensively used to investigate autonomic functions in healthy and clinical populations [9, 16]. However, pupil dynamics is also used as a proxy for neural activity associated with neural circuits beyond the circuitry responsible for pupil light and darkness reflexes [86, 87]. Given that pupillometry is an easy-to-measure technique and freely available to most modern video-based eye-tracking systems, exploring pupil responses in different behavioral tasks is crucial for developing more objective assessments to examine LLD deficits in other functions. In the current study, the pupil light and darkness reflex paradigm were used, showing the disrupted modulation of pupil and eye blink responses in LLD. These results highlight the potential of using this low-cost approach to help objectively detect LLD and evaluate the effectiveness of treatment outcomes.

### Supplementary Information

Supplementary Material 1.

Supplementary Material 2.

## Data Availability

The data used to support the findings of this study are available from the corresponding author upon reasonable request.
