# Supplementary material for: Altered pupil light and darkness reflex and eye-blink responses in late-life depression
Source: BMC Geriatr. 2024 Jun 24;24:545. doi: 10.1186/s12877-024-05034-w (PMC11194921; doi:10.1186/s12877-024-05034-w)
Supplement: Supplementary file 1 — Supplementary Material 1. [file 12877_2024_5034_MOESM1_ESM.docx]

To examine the effects of medication type on eye blink and pupil responses, similar to our previous pupillometry studies (Cherng et al., 2021; Chang et al., 2023), linear mixed models (LMMs) were used. These models allow us to consider the group effect while taking medication type and inter-participant variability into account. Our model included the dependent variable (blink and pupil indices), and group type (LLD or OLD) and the medication type (SSRI, SNRI, NDRI, TCA, MAOI, and others, as shown in Table 2) as fixed predictors. We analyzed eye blink rates, tonic pupil size variability, pupil response onset latency for the dark condition, and peak constriction velocity for the bright condition, as these results showed significant (or trending) differences between LLD and OLD. LMMs were as follows:

Model: $y=\beta_{0}+\beta_{S}+\beta_{1}Patient+\beta_{2}Med$

Where $Patient$ is patient group (LLD or OLD),$Med$ is medication type, $\beta_{S}$ is a random intercept for each participant as an individual offset, and $\beta_{0}$ is a fixed intercept, $\beta_{i}$ are the standard coefficients of the statistical model (slopes).

| **Blink = Patient + Med** | | | | | |
| --- | --- | --- | --- | --- | --- |
|  | **Beta estimate** | **Std. Error** | **t value** | **df** | **p** |
| (Intercept) | 6.63 | 1.36 | 4.86 | 51 | 1.15e-05 *** |
| Patient | 5.56 | 2.30 | 2.42 | 51 | 0.0193 * |
| Med | 0.12 | 0.69 | 0.17 | 51 | 0.86 |
| **Tonic_pupil_variability (CoV) = Patient + Med** | | | | | |
|  | **Beta estimate** | **Std. Error** | **t value** | **df** | **p** |
| (Intercept) | 9.46 | 0.66 | 14.28 | 48 | <2e-16 *** |
| Patient | -1.91 | 1.02 | -1.87 | 32 | 0.07 |
| Med | 0.09 | 0.34 | 0.27 | 51 | 0.79 |
| **Pupil response onset latency = Patient + Med** | | | | | |
|  | **Beta estimate** | **Std. Error** | **t value** | **df** | **p** |
| (Intercept) | 649.24 | 86.11 | 7.54 | 51 | 7.71e-10 *** |
| Patient | 300.72 | 144.44 | 2.08 | 34 | 0.0449 * |
| Med | -23.49 | 43.71 | -0.54 | 49 | 0.59 |
| **Peak_velocity = Patient + Med** | | | | | |
|  | **Beta estimate** | **Std. Error** | **t value** | **df** | **p** |
| (Intercept) | -12.78 | 1.17 | -10.94 | 51 | 5.6e-15 *** |
| Patient | 1.71 | 1.97 | 0.87 | 51 | 0.39 |
| Med | 0.17 | 0.59 | 0.29 | 51 | 0.78 |

SE: standard error. df: degree of freedom. t: t value. p: p value. *p < .05; **p <. 01; ***p <. 001.

As shown in the table, significant (or trending) differences between LLD and OLD in blink rates, tonic pupil size variability, and pupil response onset latencies for darkness reflex were still obtained even after taking medication type into account. These results suggest that the observed differences between LLD and OLD cannot be explained by medication type.
