# Supplementary material for: Altered pupil light and darkness reflex and eye-blink responses in late-life depression
Source: BMC Geriatr. 2024 Jun 24;24:545. doi: 10.1186/s12877-024-05034-w (PMC11194921; doi:10.1186/s12877-024-05034-w)
Supplement: Supplementary file 2 — Supplementary Material 2. [file 12877_2024_5034_MOESM2_ESM.docx]

To examine the effects of other drugs on eye blink and pupil responses, linear mixed models (LMMs) were used. Our model included the dependent variable (blink and pupil indices), as well as group type (LLD or OLD) and drug (alpha blocker, beta blocker, and benzodiazepine-BZD, as shown in Table 2) as fixed predictors. We analyzed eye blink rates, tonic pupil size variability, pupil response onset latency for the dark condition, and peak constriction velocity for the bright condition, as these results exhibited significant (or trending) differences between LLD and OLD. LMMs were as follows:

Model: $y=\beta_{0}+\beta_{S}+\beta_{1}Patient+\beta_{2}alpha+\beta_{3}beta+\beta_{4}BZD$

Where $Patient$ is patient group (LLD or OLD),$Med$ is medication type,$alpha$ is alpha blockers (yes or none), $beta$is beta blockers (yes or none), $BZD$ is benzodiazepine (yes or none), $\beta_{S}$ is a random intercept for each participant as an individual offset, and $\beta_{0}$ is a fixed intercept, $\beta_{i}$ are the standard coefficients of the statistical model (slopes).

| **Blink = Patient + alpha + beta + BZD** | | | | | |
| --- | --- | --- | --- | --- | --- |
|  | **Beta estimate** | **Std. Error** | **t value** | **df** | **p** |
| (Intercept) | 8.81 | 1.73 | 5.11 | 49 | 5.38e-06 *** |
| Patient | 5.89 | 2.01 | 2.93 | 49 | 0.00513 ** |
| alpha | -2.27 | 3.09 | -0.73 | 49 | 0.47 |
| beta | 5.16 | 2.30 | 2.25 | 49 | 0.02928 * |
| BZD | -4.80 | 2.15 | -2.23 | 49 | 0.03036 * |
| **Tonic_pupil_variability (CoV) = Patient + alpha + beta + BZD** | | | | | |
|  | **Beta estimate** | **Std. Error** | **t value** | **df** | **p** |
| (Intercept) | 10.17 | 0.89 | 11.48 | 49 | 1.87e-15 *** |
| Patient | -1.82 | 0.95 | -1.91 | 29 | 0.0659 . |
| alpha | -1.80 | 1.61 | -1.11 | 47 | 0.27 |
| beta | 0.49 | 1.15 | 0.43 | 44 | 0.67 |
| BZD | -0.84 | 1.12 | -0.75 | 48 | 0.46 |
| **Pupil response onset latency = Patient + alpha + beta + BZD** | | | | | |
|  | **Beta estimate** | **Std. Error** | **t value** | **df** | **p** |
| (Intercept) | 731.61 | 117.00 | 6.25 | 49 | 9.72e-08 *** |
| Patient | 306.68 | 133.04 | 2.31 | 26 | 0.0295 * |
| alpha | 34.75 | 210.80 | 0.17 | 44 | 0.87 |
| beta | 53.84 | 154.80 | 0.35 | 46 | 0.73 |
| BZD | -188.72 | 146.90 | -1.29 | 45 | 0.21 |
| **Peak_velocity = Patient + alpha + beta + BZD** | | | | | |
|  | **Beta estimate** | **Std. Error** | **t value** | **df** | **p** |
| (Intercept) | -12.44 | 1.57 | -7.92 | 49 | 2.55e-10 *** |
| Patient | 2.94 | 1.83 | 1.61 | 49 | 0.12 |
| alpha | 3.81 | 2.81 | 1.36 | 49 | 0.18 |
| beta | -0.20 | 2.09 | -0.09 | 49 | 0.93 |
| BZD | -1.65 | 1.96 | -0.84 | 49 | 0.41 |

SE: standard error. df: degree of freedom. t: t value. p: p value. *p < .05; **p <. 01; ***p <. 001.

As shown in the table, while beta blockers and BZD significantly affected blink rates, significant (or trending) differences between LLD and OLD in blink rates, tonic pupil size variability, and pupil response onset latencies for darkness reflex were still obtained even after taking these drugs into account. These results suggest that the observed differences between LLD and OLD cannot be explained by these drug effects.
